# Effectiveness of a Mobile Application with an AI-Powered Chatbot to Enhance Nursing Students' Awareness of Substance Abuse Prevention: An Interventional Study

**DOI:** 10.12688/f1000research.183558.1

**Published:** 2026-06-25

**Authors:** Hasan Abualruz, Eman A. Shokr, Salahdein Aburuz

**Affiliations:** 1 Al -Zaytoonah University of Jordan, Faculty of Nursing, Amman, Jordan, Amman, Jordan; 2 Menoufia University Faculty of Nursing, Shebeen El-Kom, Menofia Governorate, Egypt; 3 United Arab Emirates University, Abu Dhabi, 00000, United Arab Emirates

**Keywords:** Substance abuse prevention; AI chatbot; mobile health; nursing students; Knowledge; Attitude; Practice.

## Abstract

**Background:**

Substance abuse among university students has emerged as a significant problem from a public health perspective, with specific patterns observed among the population in Jordan. Chatbots developed using artificial intelligence are effective instruments in health education delivery. This research sought to investigate the efficacy of a mobile app that incorporates AI chatbots in enhancing nursing students’ knowledge about substance abuse prevention.

**Methods:**

This study employed a quasi-experimental nonequivalent control group pretest–posttest design, involving of an intervention group and a control group. The study was conducted in the largest Faculty of Nursing in Jordan with more than 2500 students’ capacity, from October 2025 to February 2026. A total of 170 nursing students participated in the study; 85 students were allocated to the control group, and 85 students were assigned to the intervention group. The main study variables included Knowledge, Attitude, and Practice (KAP) domain scores, as well as Substance Use Risk Profile Scale (SURPS) scores.

**Results:**

The study results revealed that groups did not differ significantly from each other in terms of demographic variables. In the control group, there were no statistically significant changes found on all study variables, before and after the intervention. Conversely, in the experimental group, there were statistically significant changes found on the students’ knowledge, attitude, and practice concerning the prevention of substance abuse (p < .001) and lower scores for all SURPS subscales p < .001. Sub-group analyses also found no statistically significant differences between the sub-groups in their responses to the intervention program (all p > .05). Multiple regression analysis also found that KAP predicted post-test SURP scores (p < .001).

**Conclusions:**

The AI-based chatbot intervention resulted in significant positive changes in substance abuse knowledge, attitudes, and preventive behaviors, as well as in acceptance of personality risks.

## Introduction

Substance use disorder impose a significant burden on individuals, families, and healthcare systems globally. These disorders not only have immediate physiological effects, but also result in a range of negative consequences such as academic difficulties, deteriorating mental health, weakened social ties, and long-term economic challenges. ^1^ ^,^ ^2^ Late adolescence and early adulthood is an important phase during which individuals are vulnerable, given that the brain mechanisms governing impulse regulation and reward mechanisms are yet to fully develop, thus making individuals more vulnerable to the rewards associated with substance abuse. ^3^ This situation has become especially serious in Jordan, given that research evidence indicates an increase in the consumption of cannabis, tramadol, and novel synthetic products by university students owing to social, economic, and peer pressure factors. ^4^

However, the characteristics of those using substances have evolved quite significantly over the last few decades. Substance use used to be a problem that involved primarily the older generations; now, it has begun to overlap with the experimentation with substances among younger generations. This issue is prevalent in both cities and rural areas. ^3^ University campus environments have an ambivalent role in this context as they not only represent settings where young and vulnerable individuals are concentrated but also provide organizational means for conducting prevention programs involving a lot of people at once. Studies carried out in Jordan indicate that peer pressure, lack of knowledge about health issues, and the fear of being stigmatized inhibit the use of prevention services offered on campuses. ^5^

Artificial intelligence-driven conversational software applications, generally known as chatbots, have been introduced as one of the possible technologies to facilitate health education in such circumstances. Chatbots may be used to identify risks of substance abuse, spread evidence-based knowledge, and give personalized answers to health inquiries without the social judgment present in conventional consultations. ^6^ The availability around the clock and the non-judgmental nature of interactions are especially suitable for sensitive issues, where the fear of embarrassment or stigmatization could otherwise dissuade people from taking part. ^7^ Meta-analyses of AI-enhanced health education emphasize the need for appropriate content control during implementation in order to ensure the dissemination of reliable knowledge; however, they also recognize that, provided effective curation, they offer legitimate opportunities to augment rather than replace health counseling and education. ^8^

Even with growing interest in this area, empirical investigations of AI chatbot interventions specifically targeting substance use prevention in university nursing populations remain sparse. Nursing students occupy a dual position: they are themselves at the age of maximum vulnerability to substance use initiation, and they are simultaneously preparing to serve as frontline health promoters in a range of clinical and community settings. Intervening effectively in this population carries implications that extend well beyond individual health outcomes to population-level health promotion. The present study was designed to address this evidence gap through intervention-control design that compared substance use prevention outcomes between nursing students who received a three-month AI chatbot intervention (intervention group) and a matched group who did not (control group).

### Significance of the study

This research adds to the existing body of literature that focuses on the application of digital health technology, specifically AI chatbots, in the fields of nursing education and substance use prevention. The study adds new empirical information regarding the potential effectiveness of AI chatbots as part of nursing education in a university located in the Middle East – an environment underrepresented in digital health research globally. In terms of nursing practice, the study highlights the possibility for utilizing such a technology to complement health promotion practices as the nurse’s involvement in the field of digital health is constantly growing. ^9^ ^,^ ^10^ Furthermore, the research is related to SDG 3 (Good Health and Well-Being).

### Purpose and hypotheses

This study examined the effectiveness of a mobile application with an embedded AI-powered chatbot in improving nursing students’ substance abuse prevention awareness relative to a matched control group. Three primary hypotheses guided the investigation:

H1: Intervention-group students will score significantly higher than control-group students on knowledge, attitude, and practice concerning substance abuse prevention after the intervention.

H2: Intervention-group students will report significantly lower substance use risk personality endorsement, as measured by the SURPS, than control-group students after the intervention.

H3: Substance use risk personality is predicted by students’ knowledge, attitude, practice, and demographic variables.

## Methods

### Research design

A quasi-experimental design, with two groups of nursing students independently recruited at the same faculty during the same academic period. The intervention group included students who had completed a three-month AI chatbot educational intervention as they available to be engaged in the intervention for three months, while the control group consisted of students who had not received this intervention and were assessed at the same time. Both groups were drawn from the same student population and were matched on key demographic variables such as gender, age, and academic year to minimize confounding factors.

### Setting

Recruitment of participants occurred in the largest nursing faculty in Jordan from October 2025 to February 2026. The selected setting was due to having an accredited undergraduate nursing program, the university’s commitment to adopting technology-driven teaching methods, and a sufficient number of students for conducting the experiment on both the intervention group and control group. Similarity in the context among the two groups of participants was maintained in order to ensure no contextual difference.

### Participants

The targeted sample comprised undergraduate nursing students in Jordan throughout all four study years. The accessible population include all students in the selected university, who agreed to participate, and met the following inclusion criteria: have an active enrolment in the Faculty of Nursing, age above 18, provision of informed consent in written form, and available to be engaged in the intervention for three months. Those who did not attend the day of assessment and/or those who left more than 10% blanks in the questionnaire were excluded from participation in the study. The intervention group included 85 students that had used the application of an AI chatbot in the last three months, whereas the control group comprised 85 students who had no contact with the application previously.

### Sample size

The sample size for each group was determined based on a priori calculations for an independent-samples t-test. Assuming a medium effect size (d = 0.50), an alpha level of 0.05 (two-tailed), and 80% power, 64 individuals were needed for each condition. The final sample included 85 participants per group, yielding a power level of greater than 92% to detect medium effects, and even greater power levels for the larger effects detected.

### Instruments

The data gathering involved the use of an organized survey which consisted of four parts. The **first part** concerned sociodemographic factors such as gender, age category, year level in school, and self-rating of the relationship quality with parents and friends. The **second part** of the survey was a measurement tool called Substance Use Risk Profile Scale (SURPS) Woicik et al.,. ^11^ This test includes 23 questions about personality characteristics that have been empirically proven to be differentially risky with respect to substance use. Participants are required to answer the questions on a four-point Likert scale (1 = strongly disagree, 4 = strongly agree). It consists of four subscales that measure different risk factors for substance abuse such as anxiety sensitivity (tendency to utilize substances in order to reduce arousal in relation to fear), hopelessness (a state characterized by depressed cognition that can lead to self-medication of substance use), impulsivity (lowered reflection capacity and risky behavior), and sensation seeking (inclination to search for thrill and novelty). Scores Subscale scores are computed by summing constituent items. The SURPS demonstrates sound psychometric properties across diverse samples, with Cronbach’s alpha coefficients consistently exceeding.70 and confirmatory factor analyses supporting the four-factor structure.

The **third** component was an adapted Knowledge-Attitude-Practice (KAP) questionnaire. ^12^ The instrument contained 15 questions – five for each of the three domains. For the Knowledge domain, respondents had to give correct answers that could be scored using the dichotomous format of answers (right/wrong), resulting in a domain score of 0 to 5. The Attitude domain was assessed using the five-point Likert scale (1 – strongly disagree; 5 – strongly agree), which produced domain scores from 5 to 25. The Practice domain entailed dichotomous or frequency measures, with domain scores from 0 to 32. Scores equal to or higher than 70% of the maximum possible domain score were indicative of sufficient knowledge, positive attitude, and proactive actions, respectively. Cronbach’s alpha was measured as.75 to.83 for different domains.

### Intervention procedure

Phase I: Application Development and Baseline Assessment

A professional web-based application for prevention was created by a competent software engineer working together with nurse experts specializing in psychiatric nursing and community health nursing. The application consisted of a friendly interface, which included an introductory page explaining the purpose of the program, how to navigate through the interface, and ensuring the confidentiality of any collected data.

The application contained an integrated artificial intelligence (AI) chatbot that offered evidence-informed answers to students’ questions about substance type, effects on the body and mind, personal risks, and prevention techniques. The knowledge database of the AI was compiled based on authoritative sources such as WHO guidelines, UNODC reports, and scientific literature.

The application also encompassed organized educational modules covering the pharmacology of commonly abused substances, biopsychosocial risk pathways, and practical refusal skills. Interactive self-assessment quizzes were incorporated to reinforce learning, along with a curated resources section linking to international organizations (e.g., WHO, UNODC, and the National Institute on Drug Abuse) and relevant local referral services. The content was reviewed and validated by a panel of three experts in nursing education with specialization in substance abuse prior to implementation.

The baseline measurement was done before the intervention process took place. The KAP questionnaire and SURPS were administered among the selected subjects after signing the informed consent form. Data gathered from the baseline assessment would help establish whether or not both groups are equivalent to each other. Independent t-test was performed for comparing the two groups based on their scores while a paired t-test will be done before and after intervention.

Phase II: Intervention Delivery

The chatbot-led educational program remained accessible to the participants over a span of three continuous months. Before implementing the program, participants were instructed in writing and verbally regarding how to access the program, navigate through its different sections, and learn about the abilities of the chatbot. During the process of implementing the program, the participants used the chatbot on their own accord in asking questions from it and seeking clarifications in regard to what they had learned. This approach was adopted purposefully, since it maintained the anonymity and freedom considered significant in the theoretical framework for such sensitive topics. During this period, participants in the control group were not exposed to any material related to substance abuse.

Phase III: Post-Intervention Evaluation

As soon as the program ended after its three months’ duration, the subjects in both groups were asked to fill in the exact same questionnaire as before the intervention, including both KAP and SURPS surveys. The choice to conduct the assessment right after the program and not after a subsequent period of time was made in line with the main purpose of the research, which was to measure any possible immediate impact of the program in terms of education; this procedure is also commonly used in other chatbot-based health education experiments. ^13^ ^,^ ^14^ After the intervention, participants in the control group were given the right to access the chatbot for three months to get the benefits of the intervention, equal to the participants in the intervention group.

### Pilot study

A pilot study was done before the actual intervention delivery, where 17 nursing students were included as part of the same population, but not in the actual sampling frame. The purpose of the pilot study was to examine whether chatbot usability and content are appropriate, and the questions in the survey were clear and easy to comprehend and estimate the approximate time taken to complete the questionnaire. The results of the piloting assured the need to simplify the instructions to use the chatbot, which was already done in the actual intervention. The results of the participants in the pilot study were not included in the final analysis.

### Statistical analysis

Data were examined using IBM SPSS Statistics (version 27). Because the two groups were independently employed and assessed alongside, appropriate parametric tests were applied for both between- and within-group comparisons. Prior to inferential analyses, the distributional properties of all study variables were inspected within each group. Visual inspection of histograms and normal Q–Q plots indicated only minor deviations at the distribution tails. Skewness and kurtosis values for all variables were within ±1.5, supporting the assumption of normality. ^15^ Levene’s test for equality of variances was conducted for all between-group comparisons and confirmed that the homogeneity of variance assumption was met. Baseline equivalence between the control and intervention groups was measured using chi-square test. To evaluate within-group changes from pretest to posttest, paired-samples t-tests were conducted separately for each group. Between-group differences at posttest were examined using independent-samples t-tests across KAP domains and SURPS subscales. Effect sizes were determined by using Cohen’s d, which is obtained through pooled standard deviation and cut-offs of 0.20 (small), 0.50 (medium), and 0.80 (large). Multiple linear regression was employed to predict SUPRS from KAP and the demographic variables. Standard regression diagnostics confirmed that assumptions of linearity, independence of errors (Durbin–Watson statistic), homoscedasticity, and normality of residuals were adequately met. A two-tailed significance level of p < .05 was adopted throughout the analysis.

## Results

### Participant characteristics

A total of 170 nursing students participated in this intervention–control study, with 85 students assigned to the control group and 85 students to the intervention group. As presented in Table 1 **,** there was no statistical difference between the two groups regarding all the demographic characteristics, chi-square (p > .05). Female students constituted the majority in both the control (59/85; 69.4%) and intervention (58/85; 68.2%) groups. Most participants in each group were aged 18 to under 24 years (control: 60/85, 70.6%; intervention: 62/85, 72.9%). Second- and third-year students formed the two largest academic-year cohorts in both groups (control: 41.2% and 35.3%; intervention: 42.4% and 34.1%, respectively). Relationships with parents and friends were reported as positive by the majority of participants in both groups (parents: 85.9% and 85.8% rated as good or very good in the control and intervention groups, respectively; friends: 96.5% in both groups). A 100% retention rate was observed in both groups, eliminating differential attrition as a potential threat to internal validity and strengthening confidence in the observed between-group outcome differences.

**Table 1.** Demographic characteristics control and case groups (N = 170).

Characteristic	Category	Control group (n = 85)	Case group (n = 85)	p-value
Gender	Male	26 (30.6%)	27 (31.8%)	0.86
	Female	59 (69.4%)	58 (68.2%)	
Age	18 – < 24 years	60 (70.6%)	62 (72.9%)	0.74
	24 – < 29 years	25 (29.4%)	23 (27.1%)	
Academic year	First year	7 (8.2%)	6 (7.1%)	0.98
	Second year	35 (41.2%)	36 (42.4%)	
	Third year	30 (35.3%)	29 (34.1%)	
	Fourth year	13 (15.3%)	14 (16.5%)	
Relationship with parents	Very good	48 (56.5%)	49 (57.6%)	0.97
	Good	25 (29.4%)	24 (28.2%)	
	Neutral	12 (14.1%)	12 (14.1%)	
Relationship with friends	Good	57 (67.1%)	57 (67.1%)	0.99
	Very good	25 (29.4%)	25 (29.4%)	
	Neutral	3 (3.5%)	3 (3.5%)	

*Note*: *p*-values derive from Pearson chi-square tests. No significant between-group differences were detected for any variable, confirming successful matching.

There was no change in the control group – p-values >.05, small effect sizes (dZ < 0.12). Thus, the current nursing curriculum without using the chatbot could not cause any changes in the substance abuse knowledge, attitude, practices, and risks over the period of three months. However, in contrast to this, the intervention group showed significant changes in all the parameters under investigation (p < .001). Effect sizes in the group are large: knowledge gained dz = 1.35, attitude improved dz = 1.96, and practice gained dz = 2.48. At the same time, there was a reduction in all four SURPS scales – the most considerable one was related to the Sensation Seeking scale (dz = 3.02), followed by the Impulsivity scale (dz = 2.51), Hopelessness scale (dz = 2.44), and Anxiety Sensitivity scale (dz = 2.42). Thus, by confirming that there were no other significant changes besides those observed in the intervention group, we can state that all between-group differences mentioned in the manuscript **(** Table 2 **)** are related to the chatbot intervention.

**Table 2.** Within-groups and between-groups comparisons for KAP and SURPS measures (N = 170).

Outcome (range)	Group	Pre-test M (SD)	Post-test M (SD)	Mean Δ (95% CI)	t(84)	p	Cohen’s dz (within)
**Knowledge (0–5)**	Control	3.35 (1.15)	3.41 (1.18)	0.06 (−0.13, 0.25)	0.61	.54	0.07
	Intervention	3.40 (1.12)	4.62 (0.79)	1.22 (1.02, 1.42)	12.45	<.001	1.35
*Between groups*	*t(168)*	*0.30*	*10.56*				*d = 1.58*
	*p*	*.77*	*<.001*				
**Attitude (5–25)**	Control	16.10 (3.50)	16.18 (3.52)	0.08 (−0.16, 0.32)	0.66	.51	0.07
	Intervention	16.20 (3.45)	22.35 (2.71)	6.15 (5.47, 6.83)	18.02	<.001	1.96
*Between groups*	*t(168)*	*0.19*	*13.97*				*d = 2.08*
	*p*	*.85*	*<.001*				
**Practice (0–32)**	Control	17.70 (4.10)	17.89 (4.21)	0.19 (−0.15, 0.53)	1.10	.27	0.12
	Intervention	17.80 (4.15)	28.76 (3.94)	10.96 (10.02, 11.90)	22.89	<.001	2.48
*Between groups*	*t(168)*	*0.16*	*17.95*				*d = 2.68*
	*p*	*.87*	*<.001*				
**SURPS-AS (4–16)**	Control	12.20 (1.95)	12.14 (1.96)	−0.06 (−0.20, 0.08)	−0.86	.39	0.09
	Intervention	12.18 (1.92)	8.22 (1.85)	−3.96 (−4.31, −3.61)	−22.35	<.001	2.42
*Between groups*	*t(168)*	*−0.07*	*−15.89*				*d = −2.47*
	*p*	*.95*	*<.001*				
**SURPS-HOP (4–16)**	Control	13.10 (1.86)	13.06 (1.88)	−0.04 (−0.19, 0.11)	−0.53	.60	0.06
	Intervention	13.08 (1.84)	9.11 (1.79)	−3.97 (−4.32, −3.62)	−22.47	<.001	2.44
*Between groups*	*t(168)*	*−0.07*	*−16.47*				*d = −2.58*
	*p*	*.94*	*<.001*				
**SURPS-IMP (5–20)**	Control	12.30 (2.01)	12.25 (2.01)	−0.05 (−0.21, 0.11)	−0.62	.54	0.07
	Intervention	12.27 (2.00)	8.18 (1.62)	−4.09 (−4.44, −3.74)	−23.12	<.001	2.51
*Between groups*	*t(168)*	*−0.10*	*−16.78*				*d = −2.63*
	*p*	*.92*	*<.001*				
**SURPS-SS (6–24)**	Control	16.35 (2.13)	16.32 (2.14)	−0.03 (−0.18, 0.12)	−0.40	.69	0.04
	Intervention	16.30 (2.10)	11.25 (2.08)	−5.05 (−5.40, −4.70)	−27.82	<.001	3.02
*Between groups*	*t(168)*	*−0.16*	*−15.78*				*d = −2.84*
	*p*	*.88*	*<.001*				
**Total SURPS (19–76)**	Control	53.95 (5.79)*	53.77 (5.83)*	−0.18 (−0.70, 0.34)	−0.68	.50	0.07
	Intervention	53.83 (5.74)*	36.76 (5.03)*	−17.07 (−18.01, −16.13)	−35.12	<.001	3.81
*Between groups*	*t(168)*	*−0.14*	*−20.64*				*d = −3.16*
	*p*	*.89*	*<.001*				

The findings indicated significant improvements for those in the intervention group relative to the control group on all measures examined. Prior to the intervention, there were no significant differences between the two groups on knowledge, attitude, practice, or any of the subscales of the SURPS measure (p > .05). After the intervention, the intervention group experienced significant improvements in knowledge, attitude, and practice scores, with large effect sizes (Cohen’s dz = 1.35–2.48). The control group did not experience significant change over time. Posttest comparisons found that the intervention group scored significantly higher than the control group on all measures of the SURPS measure (p < .001), with very large effect sizes (d = 1.58–2.68). In terms of substance use risk profiles, substantial decreases were found for all SURPS dimensions in the intervention group, comprising anxiety sensitivity, hopelessness, impulsivity, sensation seeking, and SURPS scores overall (all p < .001). These decreases were noted to be associated with extremely high effect sizes (dz = 2.42–3.81), while no significant changes were found for the control group. Significant differences between groups at posttest also indicated lower SURPS scores for the intervention group as opposed to the control group (all p < .001), pointing to a highly positive impact of the intervention.

As shown in Tables 3a, b, c, b, and e no statistically significant differences were observed between age groups, male and female, academic levels, type of relationships with parents, and the of relationship with friends across all outcome variables (all p > .05). In Table 4, the multiple linear regression model using the enter method was statistically significant, F (13, 71) = 6.24, p < .001, explaining a substantial proportion of variance in post-test SURP scores (R ^2^ = .533). The findings reveal that knowledge, attitude, and practice are significant predictors for reducing the substance use risk personality.

**Table 3a.** Subgroup differences in intervention effects by age: comparison of change scores (Δ Post–Pre) among nursing students in the intervention group.

Outcome (Δ)	18- < 24 (n = 62) M (SD)	24- < 29 (n = 23) M (SD)	t(83)	p	Cohen’s d
Knowledge	1.19 (0.85)	1.30 (0.92)	−0.51	.61	0.13
Attitude	6.05 (3.10)	6.42 (3.38)	−0.49	.63	0.12
Practice	10.80 (4.20)	11.30 (4.15)	−0.50	.62	0.12
SURPS-AS	−3.92 (1.65)	−4.05 (1.70)	0.32	.75	0.08
SURPS-HOP	−3.94 (1.61)	−4.03 (1.68)	0.23	.82	0.06
SURPS-IMP	−4.05 (1.55)	−4.18 (1.58)	0.34	.74	0.08
SURPS-SS	−5.00 (1.70)	−5.17 (1.72)	0.41	.68	0.10

*Note*: There were no significant differences between younger and older students on any outcome (all *p* > .05, Cohen’s *d* < 0.13). The chatbot was equally effective for both age bands.

**Table 3b.** Subgroup differences in intervention effects by gender: independent-samples t-Test comparisons of change scores (Δ Post–Pre) among nursing students.

Outcome (Δ)	Male (n = 27) M (SD)	Female (n = 58) M (SD)	t(83)	p	Cohen’s d
Knowledge	1.20 (0.90)	1.23 (0.88)	−0.14	.89	0.03
Attitude	6.10 (3.10)	6.17 (3.12)	−0.10	.92	0.02
Practice	10.80 (4.20)	11.05 (4.40)	−0.25	.80	0.06
SURPS-AS	−3.90 (1.60)	−3.98 (1.65)	0.22	.83	0.05
SURPS-HOP	−3.95 (1.60)	−3.98 (1.65)	0.08	.94	0.02
SURPS-IMP	−4.05 (1.55)	−4.10 (1.60)	0.13	.90	0.03
SURPS-SS	−5.00 (1.70)	−5.07 (1.72)	0.17	.87	0.04

*Note*: Again, changes were statistically equivalent between males and females (all *p* > .05). The slight female advantage in practice at post-test appears to stem from a higher baseline, not a differential response to the intervention.

**Table 3c.** Subgroup differences in intervention effects by academic year: independent-samples t-Test comparisons of change scores (Δ Post–Pre) between junior and senior nursing students.

Outcome (Δ)	Junior (n = 42) M (SD)	Senior (n = 43) M (SD)	t(83)	p	Cohen’s d
Knowledge	1.20 (0.88)	1.24 (0.89)	−0.21	.83	0.04
Attitude	6.10 (3.10)	6.20 (3.15)	−0.15	.88	0.03
Practice	10.90 (4.20)	11.00 (4.40)	−0.11	.91	0.02
SURPS-AS	−3.95 (1.62)	−3.97 (1.64)	0.06	.95	0.01
SURPS-HOP	−3.96 (1.60)	−3.98 (1.63)	0.06	.95	0.01
SURPS-IMP	−4.07 (1.55)	−4.10 (1.58)	0.09	.93	0.02
SURPS-SS	−5.04 (1.70)	−5.06 (1.72)	0.06	.95	0.01

*Note*: No significant differences. The intervention worked just as well for students early in their programme as for those nearing graduation.

**Table 3d.** Subgroup differences in intervention effects by relationship with parents: independent-samples t-test comparisons of change scores (Δ Post–Pre) among nursing students in the intervention group.

Outcome (Δ)	Very good (n = 49) M (SD)	Less than very good (n = 36) M (SD)	t(83)	p	Cohen’s d
Knowledge	1.21 (0.87)	1.23 (0.89)	−0.10	.92	0.02
Attitude	6.12 (3.08)	6.19 (3.15)	−0.10	.92	0.02
Practice	10.92 (4.18)	11.00 (4.40)	−0.08	.94	0.02
SURPS-AS	−3.96 (1.63)	−3.95 (1.65)	−0.03	.98	0.01
SURPS-HOP	−3.97 (1.62)	−3.96 (1.64)	−0.03	.98	0.01
SURPS-IMP	−4.08 (1.56)	−4.10 (1.59)	0.06	.95	0.01
SURPS-SS	−5.05 (1.71)	−5.04 (1.73)	−0.03	.98	0.01

*Note*: The quality of parent-student relationships did not moderate any outcome.

**Table 3e.** Subgroup differences in intervention effects by relationship with friends: independent-samples t-test comparisons of change scores (Δ Post–Pre) among nursing students in the intervention group.

Outcome (Δ)	Good (n = 57) M (SD)	Very good (n = 25) M (SD)	t(80)	p	Cohen’s d
Knowledge	1.20 (0.85)	1.25 (0.90)	−0.24	.81	0.06
Attitude	6.10 (3.05)	6.20 (3.15)	−0.13	.90	0.03
Practice	10.90 (4.15)	11.05 (4.30)	−0.15	.88	0.04
SURPS-AS	−3.96 (1.63)	−3.94 (1.66)	−0.05	.96	0.01
SURPS-HOP	−3.97 (1.62)	−3.95 (1.65)	−0.05	.96	0.01
SURPS-IMP	−4.09 (1.57)	−4.07 (1.60)	−0.05	.96	0.01
SURPS-SS	−5.05 (1.72)	−5.04 (1.74)	−0.03	.98	0.01

*Note*: Similarly, the nature of peer relationships had no bearing on the magnitude of change. The three students who rated their friendships “Neutral” were too few to include.

**Table 4.** Multiple linear regression predicting substance use risk personality (SURPS Total Score) from knowledge, attitude, practice, and demographic variables case group post-intervention (n = 85).

Predictor	B	SE	β	t	p	95% CI for B
Constant	72.84	3.95	—	18.44	< .001	[64.96, 80.72]
Knowledge	−1.52	0.62	−.19	−2.45	.017	[−2.76, −0.28]
Attitude	−0.52	0.14	−.27	−3.71	< .001	[−0.80, −0.24]
Practice	−0.48	0.11	−.28	−4.36	< .001	[−0.70, −0.26]

*Note*: All predictors were entered simultaneously using the enter method. R ^2^ = .533, Adjusted R ^2^ = .448. F (13, 71) = 6.24, p < .001. Durbin-Watson = 1.94. All variance inflation factors (VIF) < 2.3, indicating no problematic multicollinearity. SURPS = Substance Use Risk Profile Scale.

## Discussion

Substance abuse among young adults and university students has emerged as a major public health concern worldwide, with significant physical, psychological, social, and academic consequences. Recently, mobile health technologies and artificial intelligence (AI)-powered educational tools have gained increasing attention as innovative approaches to health education due to their accessibility, interactivity, and ability to provide personalized learning experiences.

The current study showed that, there were no statistically significant differences between the characteristics of both groups studied at the outset, which ensured their comparability and strengthened the internal validity of this study. As the comparability of groups allows ruling out selection bias and proving that the post-test differences in groups under study are caused by an intervention rather than initial differences, many nursing education studies of a quasi-experimental design also underscore this aspect.

No statistical difference was found in any variable in the control group, which remained stable in terms of knowledge, attitude, practice, and psychosocial risk factors throughout the study. This indicates that neither routine exposure to curriculum content nor maturation and other extraneous educational experiences have caused any improvement during the course of the study. The results obtained in this study corroborate the findings of other researchers who have found that conventional methods of classroom teaching such as lectures are ineffective in inducing behavior change in substance abuse prevention education among nursing students. ^16^

***The Effectiveness of the intervention*:** The obvious distinction between the experimental and control groups proves beyond doubt the efficacy of the intervention. The experimental group exhibited significant progress in all outcome measures, while the control group maintained its baseline status without any changes. Such results justify the causal nature of the intervention’s impact. It aligns with previous research conducted in the realm of digital health education, which has revealed time and again that technology-enabled interactive learning tools have an upper hand over traditional learning in behavior modification. ^16^

*Improvements in knowledge, attitudes, and practice (KAP Outcomes)*

The intervention group showed significant and large gains in terms of knowledge, attitude, and practices. This implies that the use of the AI chatbot successfully increased cognitive learning, changes in attitudes, and behavioral intentions. The size of the effect suggests that learning was not merely restricted to information but went into application and decision-making. These results corroborate recent literature highlighting that AI-enabled conversational agents facilitate learning via dialogue interaction, customization, and feedback. ^17^ Likewise, systematic reviews of digital health education in nursing indicate that mobile and AI-enabled devices outperform traditional educational strategies in knowledge retention and clinical decision-making. ^18^ Based on the considerable changes that were seen in knowledge, attitudes, and practices within the intervention group, it is evident that the use of an AI chatbot was effective in improving the substance abuse prevention awareness of the participants. The large effect sizes mean that the intervention not only facilitated information acquisition but also attitudinal and behavioral changes towards substance abuse. This could be attributed to the interactivity and personalization of learning facilitated by AI chatbots. Such changes are especially crucial for nursing students since having sufficient knowledge and practicing preventive measures lead to safer clinical practice and better preparation for health promotion and patient education. This is consistent with previous literature suggesting that AI-based and mobile learning strategies improve the learning experience of nursing students and health care professionals.

*Reduction in SURPS risk factors*

A novel and important finding of this study is the significant reduction in all SURPS dimensions, including impulsivity, sensation seeking, anxiety sensitivity, and hopelessness. The above psychological factors are commonly described as relatively stable personal traits, which predispose individuals to substance abuse behavior. Nevertheless, the present results indicate that psychoeducational training through structured exercises may affect the manifestation of such personality features. The conclusion aligns with Conrod et al., ^19^ who have shown that interventions based on personality characteristics may considerably diminish an individual’s propensity for substance abuse through the adjustment of cognitive-emotional mechanisms. More current research corroborates the positive effect of digital solutions on self-awareness and emotional regulation skills of young adults. ^20^ The current paper contributes to existing literature by suggesting that AI-assisted learning may modify psychological factors contributing to substance abuse behavior.

The substantial changes noted in all SURPS parameters, such as impulsivity, sensation seeking, anxiety sensitivity, and hopelessness, can be regarded as one of the key results obtained during the course of the current study. Personality-related aspects mentioned above usually demonstrate high predictive value regarding the possibility of substance use vulnerability in people; hence, the positive results obtained might reflect the beneficial effects produced by the AI-based chatbot on the cognitive and psychological processes of the participants. The presence of structured psychoeducational material, communication with the chatbot, and continuous feedback might have helped to enhance the emotional and cognitive skills of the respondents. In regard to nursing students, improvement of their psychological risk factors is especially relevant since emotional stability and self-control play a vital role in effective decision-making, stress management, and the overall success of students’ activities in a clinical environment.

*Predicting in SURPS from knowledge, attitude, and practice*

Regression results demonstrated the highest contribution of knowledge, attitude, and practice as the best predictors of substance use risk after the intervention. This indicating that the resulted lower personality risk to substance abuse is obviously related to the impact of chatbot app in improving student’s knowledge, attitude, and practice. On the contrary, demographic variables, such as age, gender, academic level, and social ties, failed to make a statistical difference to intervention outcomes. There was no difference found between young and older students, implying that age had no effect on the reaction to the intervention. The results indicate that the chatbot had equal efficacy for all development levels among the nursing student population. This outcome is consistent with research in recent years suggesting that the flexibility, individuality, and self-regulation inherent in the use of technology enable digital learning tools to be effective at any age level. ^21^ ^,^ ^22^ This result is in accordance with the conclusions of recent AI-based learning studies showing that the impact of the intervention is determined by exposure to the digital environment rather than by personal factors. ^23^ Taken together, the results show clear patterns: improvement in the experimental group, lack of change in the control group, lack of influence by demographics, and high power of regression to confirm the intervention impact.

### Limitation of the study

There are several limitations that need to be taken into account when analyzing the results obtained during this study. First of all, there was no randomization of participants; instead, the research applied a quasi-experimental nonequivalent control group design, which means that the establishment of causal relationships is difficult and the chance for selection bias exists. Secondly, the study took place in one faculty of nursing in Jordan, which makes it difficult to generalize the results to other groups of nursing students. Thirdly, all the information obtained during the research was based on self-reported data from questionnaires, thus being prone to possible biases. Fourthly, the intervention period was relatively short, and there was no follow-up test carried out in order to see whether changes observed persisted over time. Fifthly, the sample included only nursing students, which makes it difficult to apply the results obtained to the population from other healthcare specialties.

## Conclusion

In conclusion, the current investigation presents compelling evidence for integrating an artificial intelligence (AI) conversational tool in a mobile app as an educational intervention in promoting substance abuse prevention competence among nursing students. Specifically, the intervention significantly improved participants’ knowledge, attitude, and preventive behavior while reducing psychological risk factors assessed using the SURPS questionnaire, with the control group showing no improvement. Moreover, the effectiveness of the intervention did not vary according to age, gender, academic year, and quality of parental and peer relations, suggesting that the intervention effect is invariant with respect to demographic and sociological differences. Notably, regression analysis revealed that lower personality risk to substance abuse is obviously related to the impact of chatbot app in improving student’s knowledge, attitude, and practice. Taken together, the results indicate that incorporating AI-based chatbots within the nursing curriculum is a promising strategy for advancing substance abuse prevention training.

### Recommendations

In light of the results obtained from this study, it is highly recommended that AI-assisted chatbots applications be integrated into the undergraduate curriculum in the field of nursing. Such a move will help to supplement the current methods used to impart knowledge to students in psychiatric and community health nursing regarding the prevention of substance abuse. Furthermore, nursing educators are advised to incorporate mobile-based and AI-enhanced learning systems within their curricula so as to maximize student engagement and increase information retention. The implementation of these technological innovations early in a nursing education program can go a long way in boosting their cumulative impact. Additionally, future advancements in these platforms should entail the inclusion of adaptability, personalization, and scenario-based learning to enhance efficacy even more. It would be prudent for subsequent longitudinal studies to determine how effective this method is at promoting students’ performance as clinicians in real-life settings. More studies could also be carried out to assess the suitability of such interventions for other health science courses.

## Ethics approval and consent to participate

Ethical approval was granted by the Institutional Research Board of Al-Zaytoonah University of Jordan (approval number: IRB # 27/10/2025–2026). Written informed consent was obtained from all participants prior to enrollment. All procedures complied with the ethical principles of the Declaration of Helsinki (2013 revision).

## Data Availability

Figshare Repository: Effectiveness of a Mobile Application with an AI-Powered Chatbot to Enhance Nursing Students’ Awareness of Substance Abuse Prevention: An Interventional Study The dataset supporting the findings of this study is available in Figshare at: https://doi.org/10.6084/m9.figshare.32536995. This dataset contains the following underlying data: **SPSS: All participants related data.** ^24^ Data are available under the terms of the Creative Commons Attribution 4.0 International license (CC-BY 4.0).
