# Supplementary material for: Effectiveness of a Mobile Application with an AI-Powered Chatbot to Enhance Nursing Students' Awareness of Substance Abuse Prevention: An Interventional Study
Source: F1000Res. 2026 Sep 17;15:1015. Originally published 2026 Jun 25. [Version 2] doi: 10.12688/f1000research.183558.2 (PMC13615354; doi:10.12688/f1000research.183558.2)
Supplement: Peer review history

# Reviewer report: Reviewer response for version 2

**DOI:** 10.5256/f1000research.210051.r522223

**Published:** 2026-09-25

**Authors:** Febblina Daryanes

**Affiliations:** 1 Universitas Riau, Pekanbaru, Riau, Indonesia

I have no further comments. The authors have adequately addressed the reviewers’ comments, and the revised manuscript has been substantially improved.

Is the work clearly and accurately presented and does it cite the current literature?

Partly

If applicable, is the statistical analysis and its interpretation appropriate?

Yes

Are all the source data underlying the results available to ensure full reproducibility?

Partly

Is the study design appropriate and is the work technically sound?

Partly

Are the conclusions drawn adequately supported by the results?

Partly

Are sufficient details of methods and analysis provided to allow replication by others?

Partly

Reviewer Expertise:

Biology education, educational research

I confirm that I have read this submission and believe that I have an appropriate level of expertise to confirm that it is of an acceptable scientific standard.

# Reviewer report: Reviewer response for version 2

**DOI:** 10.5256/f1000research.210051.r522224

**Published:** 2026-09-22

**Authors:** Louis Robert C. Sison

**Affiliations:** 1 Bulacan State University, Bulacan, Philippines

The authors have addressed all comments and suggestions given in the previous review. I have no further comments to make. Thank you.

Is the work clearly and accurately presented and does it cite the current literature?

Partly

If applicable, is the statistical analysis and its interpretation appropriate?

Partly

Are all the source data underlying the results available to ensure full reproducibility?

Yes

Is the study design appropriate and is the work technically sound?

Partly

Are the conclusions drawn adequately supported by the results?

Partly

Are sufficient details of methods and analysis provided to allow replication by others?

Partly

Reviewer Expertise:

Mathematics Education, Pre-service Teacher Education

I confirm that I have read this submission and believe that I have an appropriate level of expertise to confirm that it is of an acceptable scientific standard.

# Reviewer report: Reviewer response for version 1

**DOI:** 10.5256/f1000research.202613.r511064

**Published:** 2026-09-04

**Authors:** Louis Robert C. Sison

**Affiliations:** 1 Bulacan State University, Bulacan, Philippines

1. The manuscript establishes the public-health relevance of substance abuse among university students, provides specific contextual justification for Jordan, and explains why nursing students represent an important target population. Importantly, the authors identify an empirical gap, which studies of AI-chatbot interventions specifically targeting substance-use prevention among university nursing students remain limited. Moreover, a good topical currency is demonstrated by the paper on multiple topics on substance-use disorders, digital health, AI-supported nursing education, and chatbot applications. 

 However, the conceptual progression from the identified problem to the proposed intervention needs greater theoretical depth. The manuscript discusses the technological advances of chatbots but does not clearly establish a theoretical framework explaining how chatbot interaction is expected to produce changes in knowledge, attitudes, practices, and especially relatively stable personality-risk traits measured by SURPS. ***It is recommended to strengthen the theoretical pathway linking the intervention components to each outcome.***

*Nevertheless, **the manuscript should more clearly explain the mechanism **by which chatbot-based education is expected to influence not only knowledge, attitudes, and practices but also the relatively stable personality-risk dimensions measured by the SURPS.* Terminology such as awareness, knowledge, practice, behavior, competence, and personality risk should also be used more consistently because these represent conceptually distinct outcomes. The discussion would benefit from a more critical synthesis of the literature rather than relying predominantly on studies supportive of digital interventions.

 2. The quasi-experimental pretest–posttest design with a control group is appropriate for evaluating the intervention. The use of baseline measures, comparable groups, and a priori sample-size calculation are strengths. *However, the lack of randomization should be acknowledged as a limitation when making causal claims. **In addition, the intervention procedures need clearer reporting. The manuscript should specify how often students used the chatbot, how long they interacted with it, and whether their participation was monitored.** These details are important for assessing intervention fidelity and for allowing other researchers to replicate the study.*

 3. The manuscript provides useful information regarding participant eligibility, sample size, the KAP and SURPS measures, application development, intervention duration, and the general phases of implementation. The chatbot content was reportedly based on authoritative sources and reviewed by nursing experts. Nevertheless, replication would require substantially more operational detail. ***The authors should report the exact nature or model of the AI chatbot***, the structure of its knowledge base, safeguards against inaccurate responses, sample prompts or interactions, educational module content, expected frequency of use, monitoring procedures, and intervention-fidelity measures. ***The adapted KAP questionnaire should also be provided or made accessible, together with its scoring procedure and evidence of validity and reliability, specifically within the present study population. *** These additions are necessary for another research team to reproduce both the intervention and its evaluation.

 4. The statistical analyses are generally appropriate and clearly reported. However, since the study used a pretest–posttest design with a control group, ***the authors may consider using ANCOVA or a Group × Time analysis to more directly compare the changes between groups while accounting for baseline scores. *** This would strengthen the evidence for the intervention effect.

 The reported effect sizes, especially for the SURPS outcomes, are also very large. It would be helpful for the authors to discuss these findings more cautiously and consider possible influences such as self-report bias or other uncontrolled factors.

 5. The conclusions are generally supported by the results. However, some statements may be expressed with greater caution because the study employed a nonrandomized quasi-experimental design. Instead of stating that the intervention “proved” its effectiveness, ***the authors may say that the findings support the chatbot intervention's potential effectiveness***. It would also be helpful to clarify that the reported changes in practice and SURPS scores are based on self-reported measures and should be interpreted with this limitation in mind.

Is the work clearly and accurately presented and does it cite the current literature?

Partly

If applicable, is the statistical analysis and its interpretation appropriate?

Partly

Are all the source data underlying the results available to ensure full reproducibility?

Yes

Is the study design appropriate and is the work technically sound?

Partly

Are the conclusions drawn adequately supported by the results?

Partly

Are sufficient details of methods and analysis provided to allow replication by others?

Partly

Reviewer Expertise:

Mathematics Education, Pre-service Teacher Education

I confirm that I have read this submission and believe that I have an appropriate level of expertise to confirm that it is of an acceptable scientific standard, however I have significant reservations, as outlined above.

# Response

**Published:** 2026-09-05

**Authors:** Eman Shokr

**Affiliations:** Menoufia University Faculty of Nursing, Shebeen El-Kom, Menofia Governorate, Egypt

**Reviewer 2**

 1. The manuscript establishes the public-health relevance of substance abuse among university students, provides specific contextual justification for Jordan, and explains why nursing students represent an important target population. Importantly, the authors identify an empirical gap, which studies of AI chatbot interventions specifically targeting substance-use prevention among university nursing students remain limited. Moreover, a good topical currency is demonstrated by the paper on multiple topics on substance-use disorders, digital health, AI-supported nursing education, and chatbot applications.

 Thank you for this positive and encouraging assessment of our manuscript. We also appreciate the recognition of the identified empirical gap regarding the limited evidence on AI-chatbot interventions specifically designed to address substance-use prevention among university nursing students.

 - However, the conceptual progression from the identified problem to the proposed intervention needs greater theoretical depth. The manuscript discusses the technological advances of chatbots but does not clearly establish a theoretical framework explaining how chatbot interaction is expected to produce changes in knowledge, attitudes, practices, and especially relatively stable personality-risk traits measured by SURPS. It is recommended to strengthen the theoretical pathway linking the intervention components to each outcome.

 Thank you for this important and constructive comment. we have substantially revised and strengthened the Theoretical Framework section to provide a clearer theoretical pathway linking the intervention components to the expected outcomes.

 - Nevertheless, the manuscript should more clearly explain the mechanism by which chatbot-based education is expected to influence not only knowledge, attitudes, and practices but also the relatively stable personality-risk dimensions measured by the SURPS. Terminology such as awareness, knowledge, practice, behavior, competence, and personality risk should also be used more consistently because these represent conceptually distinct outcomes. The discussion would benefit from a more critical synthesis of the literature rather than relying predominantly on studies supportive of digital interventions.

 Thank you for this valuable and constructive comment. We have carefully revised the manuscript to address the concerns regarding the mechanisms underlying the intervention, the consistency of terminology, and the critical synthesis of the existing literature.

 2. The quasi-experimental pretest–posttest design with a control group is appropriate for evaluating the intervention. The use of baseline measures, comparable groups, and a priori sample-size calculation are strengths. *However, the lack of randomization should be acknowledged as a limitation when making causal claims. **In addition, the intervention procedures need clearer reporting. The manuscript should specify how often students used the chatbot, how long they interacted with it, and whether their participation was monitored.** These details are important for assessing intervention fidelity and for allowing other researchers to replicate the study.*

 Thank you for this valuable and constructive comment. We have clarified in the revised manuscript that, although the use of a control group, baseline assessment, comparable groups, and an a priori sample-size calculation strengthened the study design, the absence of randomization limits the ability to establish definitive causal relationships and may introduce the possibility of selection bias. The interpretation of the intervention effects has therefore been revised to avoid overstating causality.

 3. The manuscript provides useful information regarding participant eligibility, sample size, the KAP and SURPS measures, application development, intervention duration, and the general phases of implementation. The chatbot content was reportedly based on authoritative sources and reviewed by nursing experts. Nevertheless, replication would require substantially more operational detail. The authors should report the exact nature or model of the AI chatbot, the structure of its knowledge base, safeguards against inaccurate responses, sample prompts or interactions, educational module content, expected frequency of use, monitoring procedures, and intervention-fidelity measures. The adapted KAP questionnaire should also be provided or made accessible, together with its scoring procedure and evidence of validity and reliability, specifically within the present study population. These additions are necessary for another research team to reproduce both the intervention and its evaluation.

 Thank you for this valuable and constructive comment. We expanded the Methods section to provide a more detailed description of the chatbot architecture, knowledge base, educational content, monitoring procedures, intervention fidelity, and the adapted KAP questionnaire.

 4. The statistical analyses are generally appropriate and clearly reported. However, since the study used a pretest–posttest design with a control group, the authors may consider using ANCOVA or a Group × Time analysis to more directly compare the changes between groups while accounting for baseline scores. This would strengthen the evidence for the intervention effect.

 The reported effect sizes, especially for the SURPS outcomes, are also very large. It would be helpful for the authors to discuss these findings more cautiously and consider possible influences such as self-report bias or other uncontrolled factors.

 Thank you for this valuable suggestion. We agree that ANCOVA or a Group × Time analysis could provide a more direct assessment of between-group differences in change over time. However, these analyses were not prespecified in the original statistical analysis plan. Therefore, we have approved the absence of such an analysis as a limitation of the present study. We have also added a recommendation that future studies incorporate more advanced longitudinal analytical approaches to provide a more comprehensive assessment of differential changes between intervention and control groups over time.

 5. The conclusions are generally supported by the results. However, some statements may be expressed with greater caution because the study employed a nonrandomized quasi-experimental design. Instead of stating that the intervention “proved” its effectiveness, ***the authors may say that the findings support the chatbot intervention's potential effectiveness***. It would also be helpful to clarify that the reported changes in practice and SURPS scores are based on self-reported measures and should be interpreted with this limitation in mind.

 Thank you for this valuable comment. Accordingly, the Conclusions section has been revised to avoid causal or overly definitive language. Statements suggesting that the intervention “proved” its effectiveness have been replaced with more cautious wording indicating that the findings support the potential effectiveness of the AI-powered chatbot intervention in improving nursing students’ substance abuse prevention-related outcomes.

# Reviewer report: Reviewer response for version 1

**DOI:** 10.5256/f1000research.202613.r511069

**Published:** 2026-08-31

**Authors:** Febblina Daryanes

**Affiliations:** 1 Universitas Riau, Pekanbaru, Riau, Indonesia

1. The theoretical foundation needs to be strengthened, and the literature supporting the research framework should be further developed and discussed in greater depth.

 2. The overall research design is appropriate; however, the details of the intervention, particularly Phase II, need to be clarified to allow the appropriateness and implementation of the intervention to be assessed more convincingly.

 3. The intervention procedures are not described in sufficient detail, and further clarification is needed regarding the validity and reliability of the instruments used in the study.

 4. If the raw data are not currently available or the procedures for accessing the data are unclear, the authors should clarify the data availability statement and provide the necessary supporting data to facilitate reproducibility, while ensuring compliance with relevant ethical and privacy requirements.

 5. The conclusions are generally supported by the reported findings. However, the implications of the study should be articulated more clearly, and some interpretations should be presented more cautiously to ensure that they remain consistent with the evidence obtained.

Is the work clearly and accurately presented and does it cite the current literature?

Partly

If applicable, is the statistical analysis and its interpretation appropriate?

Yes

Are all the source data underlying the results available to ensure full reproducibility?

Partly

Is the study design appropriate and is the work technically sound?

Partly

Are the conclusions drawn adequately supported by the results?

Partly

Are sufficient details of methods and analysis provided to allow replication by others?

Partly

Reviewer Expertise:

Biology education, educational research

I confirm that I have read this submission and believe that I have an appropriate level of expertise to confirm that it is of an acceptable scientific standard, however I have significant reservations, as outlined above.

# Response

**Published:** 2026-09-05

**Authors:** Eman Shokr

**Affiliations:** Menoufia University Faculty of Nursing, Shebeen El-Kom, Menofia Governorate, Egypt

**Reviewer 1**                                                                                 

 1. The theoretical foundation needs to be strengthened, and the literature supporting the research framework should be further developed and discussed in greater depth.

 Thank you for this valuable and constructive comment. We revised and strengthened the introduction to provide a clearer theoretical rationale for the intervention and to establish a stronger connection between the theoretical framework, the intervention components, and the expected substance-use prevention outcomes. Specifically, we have now explicitly grounded the intervention primarily in the Health Belief Model (HBM) and incorporated relevant principles of Social Cognitive Theory (SCT) to provide a more comprehensive behavioral rationale.

 2. The overall research design is appropriate; however, the details of the intervention, particularly Phase II, need to be clarified to allow the appropriateness and implementation of the intervention to be assessed more convincingly.

 Thank you for this valuable comment. We have carefully revised the manuscript to clarify the intervention, with particular emphasis on **Phase II**. Additional details regarding the content, procedures, implementation, frequency, duration, and delivery of the intervention have been added to the revised manuscript.

 3. The intervention procedures are not described in sufficient detail, and further clarification is needed regarding the validity and reliability of the instruments used in the study.

 Thank you for this important comment. We have thoroughly revised the manuscript to provide a more detailed description of the intervention procedures, including the content, delivery method, frequency, duration, and implementation of each intervention phase and provided additional information regarding their validity and reliability.

 4. If the raw data are not currently available or the procedures for accessing the data are unclear, the authors should clarify the data availability statement and provide the necessary supporting data to facilitate reproducibility while ensuring compliance with relevant ethical and privacy requirements.

 Thank you for this important comment. We have clarified the **Data Availability Statement** in the revised manuscript to ensure transparency and facilitate reproducibility. The complete dataset underlying the findings of this study is publicly available in the **Figshare Repository** and can be accessed through the provided DOI: **https://doi.org/10.6084/m9.figshare.32536995** .

 5. The conclusions are generally supported by the reported findings. However, the implications of the study should be articulated more clearly, and some interpretations should be presented more cautiously to ensure that they remain consistent with the evidence obtained.

 Thank you for this valuable comment. We have carefully revised the Conclusion and Implications sections to provide a clearer articulation of the implications of the study findings for nursing education and substance abuse prevention. We have also moderated some of the interpretations to ensure that all conclusions remain appropriately aligned with the evidence obtained from the study.
